# Dichloroacetate potentiates tamoxifen-induced cell death in breast cancer cells via downregulation of the epidermal growth factor receptor

**DOI:** 10.18632/oncotarget.10999

**Published:** 2016-08-01

**Authors:** Sang Hyeok Woo, Sung-Keum Seo, Yoonhwa Park, Eun-Kyu Kim, Min-Ki Seong, Hyun-Ah Kim, Jie-Young Song, Sang-Gu Hwang, Jin Kyung Lee, Woo Chul Noh, In-Chul Park

**Affiliations:** ^1^ Division of Radiation Cancer Research, Korea Institute of Radiological and Medical Sciences, Nowon-gu, Seoul, 01812, Republic of Korea; ^2^ School of Life Science and Biotechnology, Korea University, Seongbuk-gu, Seoul, 02841, Republic of Korea; ^3^ Department of Surgery, Breast Cancer Center, Seoul National University Bundang Hospital, Seoul National University College of Medicine, Bundang-gu, Seongnam, 13620, Republic of Korea; ^4^ Department of Surgery, Korea Cancer Center Hospital, Korea Institute of Radiological and Medical Sciences, Nowon-gu, Seoul, 01812, Republic of Korea; ^5^ KIRAMS Radiation Biobank, Korea Institute of Radiological and Medical Sciences, Nowon-gu, Seoul, 01812, Republic of Korea

**Keywords:** tamoxifen, breast cancer, dichloroacetate, epidermal growth factor receptor, pyruvate dehydrogenase kinase

## Abstract

Metabolic reprogramming in cancer cells has recently been recognized as an essential hallmark of neoplasia. In this context, metabolic alterations represent an attractive therapeutic target, and encouraging results with drugs targeting various metabolic processes have been obtained in preclinical studies. Recently, several studies have suggested that dichloroacetate (DCA), a specific pyruvate dehydrogenase kinase inhibitor, may be a potential anticancer drug in a large number of diverse tumors. However, the precise mechanism is not fully understood, which is important for the use of DCA in cancer treatment. In the present study, we found that DCA sensitized MCF7 breast cancer cells to tamoxifen-induced cell death by decreasing epidermal growth factor receptor (EGFR) expression. The downregulation of EGFR was caused by degradation of the protein. Furthermore, p38 mitogen-activated protein kinase played an important role in DCA/tamoxifen-induced EGFR degradation. Finally, DCA also promoted comparable tamoxifen-induced cell death in tamoxifen-resistant MCF7 cells, which were established by long-term treatment with tamoxifen. In summary, our results suggest that DCA is an attractive potential drug that sensitizes cells to tamoxifen-induced cell death and overcome tamoxifen resistance via downregulation of EGFR expression in breast cancer cells.

## INTRODUCTION

Proliferating cancer cells have considerably different metabolic requirements compared to most normal differentiated cells. For example, to support rapid cell growth and proliferation, cancer cells differentially alter metabolic flux compared to the surrounding tissue to provide sufficient bioenergetics and biosynthetic intermediates. A well-known phenomenon observed in most cancer cells is a shift to aerobic glycolysis, regardless of oxygen supply, which is termed the “Warburg effect”, in which pyruvate is directly converted to lactic acid instead of entering the citric acid cycle [1]. As all cancer cells are dependent on this change in metabolism, these altered pathways represent attractive therapeutic targets [2]. Efforts have been made to target reprogrammed metabolism alone or in combination with cancer chemotherapy both in preclinical and clinical studies [3]. Interestingly, this cancer-specific metabolic remodeling is reversed by dichloroacetate (DCA), a mitochondria-targeting small molecule that can penetrate most tissues after oral administration [4]. It specifically inhibits pyruvate dehydrogenase kinase (PDK), a member of the kinase family, leading to reactivation of pyruvate dehydrogenase (PDH), a key enzyme that shifts the flux of pyruvate into the mitochondria to promote glucose oxidation instead of glycolysis [4]. Although DCA has recently been evaluated in several preclinical cancer trials [5], the responses of cancer cells to DCA treatment, which determine whether DCA will provide clinical benefit in cancer treatment, have not been fully elucidated.

More than 70% of breast cancers express the estrogen receptor (ER) and depend on estrogen to drive tumor growth and progression [6]. Thus, endocrine therapy should be considered complementary to surgery in the majority of patients, as it induces tumor remission and provides consistent clinical benefits. The anti-estrogen drug tamoxifen is the most commonly used treatment for patients with ER-positive breast cancer in both the early and the advanced/metastatic stages [7]. As an adjuvant therapy in early breast cancer, tamoxifen improves overall survival, and its widespread use is believed to have significantly contributed to the reduction in breast cancer mortality observed over the last decade [8]. Despite the obvious benefits of tamoxifen treatment in breast cancer, almost all patients with metastatic disease and as many as 25% of patients receiving adjuvant tamoxifen eventually relapse and die due to the disease [9, 10]. The biological mechanisms underlying intrinsic (*de novo*) and acquired resistance to tamoxifen are therefore of considerable clinical importance. A better understanding of these mechanisms may suggest new strategies to overcome tamoxifen resistance and improve the treatment of breast cancer.

In the present study, we demonstrated that the expression of EGFR in breast cancer cells was decreased by treatment with DCA. A combination of DCA and tamoxifen further lowered the EGFR levels. We showed that DCA enhanced the cytotoxicity of tamoxifen to breast cancer cells by inhibiting EGFR expression. In addition, DCA sensitized the tamoxifen-resistant MCF7 cells to tamoxifen via EFGR downregulation. These results suggest the potential utilization of DCA in breast cancer treatment by attenuating the EGFR signaling pathway.

## RESULTS

### Inhibition of PDK downregulates EGFR and enhances tamoxifen-induced cell death in breast cancer cells

**Figure 1:**
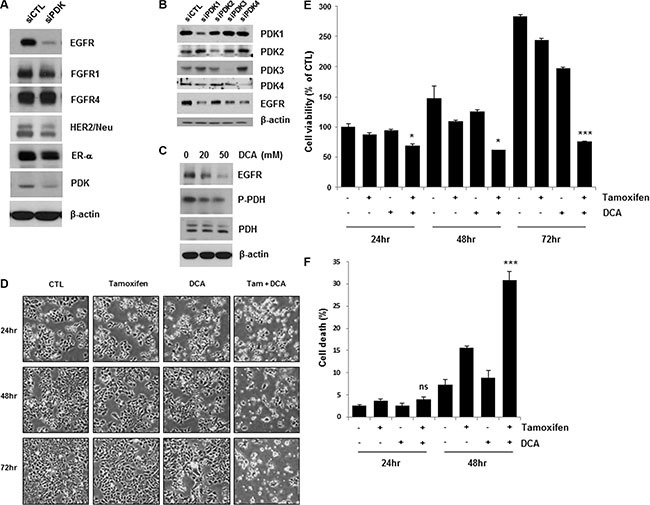
Inhibition of PDK enhanced tamoxifen-induced cell death in MCF7 cells (**A** and **C**) MCF7 cells were treated with siRNA against PDK (A) or 20–50 μM DCA (C) for 36 h, and the cell lysates were subjected to Western blotting. The blot is representative of two independent experiments. B. MCF7 cells were treated with siRNA against PDK1, PDK2, PDK3, or PDK4 for 48 h, and the cell lysates were subjected to Western blotting. (**D** and **E**) MCF7 cells were treated with or without 10 μM tamoxifen and/or 20 mM DCA for 48–72 h. The cell morphological changes (D) were observed under an inverted microscope, and the images are representative of three independent experiments. Cell viability (E) was assessed using an MTT assay. Data are presented as the mean of triplicate samples, and error bars reflect the SD. (**F**) MCF7 cells were treated with or without 10 μM tamoxifen or/and 20 mM DCA for 24 and 48 h. Cell death was evaluated by flow cytometry after Annexin V and PI staining. Data are presented as the mean of triplicate samples, and error bars reflect the SD. *p < 0.05;***p < 0.001 compared to untreated. ns, nonsignificant.

Recent studies have shown that targeting PDK by a PDK inhibitor, such as DCA, shifts the cancer cell metabolism from glycolysis to oxidative phosphorylation by dephosphorylating mitochondrial pyruvate dehydrogenase [4, 11]. To elucidate how growth factor and kinase signaling pathways in conjunction with PDK regulate the Warburg effect in breast cancer, we examined the expression levels of several growth factor receptors in PDK knockdown MCF7 cells. Interestingly, the depletion of PDK by siRNA treatment downregulated EGFR, in contrast with no or marginal effects on other growth factor receptors (Figure 1A). Four PDK isoenzymes (PDK1, PDK2, PDK3, PDK4) have been identified in mammalian tissues [12]. To confirm the downregulation of EGFR by siPDK treatment, we explored EGFR expression in the cells treated with siRNA against each isoform of PDK. Each siRNA treatment only abolished the expression of the targeted PDK, however, all of them caused the downregulation of EGFR, suggesting that PDK may not regulate EGFR expression in an isoform-specific manner (Figure 1B). Consistent with these results, DCA also reduced EGFR levels in a dose-dependent manner (Figure 1C). When we analyzed lactate concentration in the culture media, there was no significant change of lactate concentration by treatment with tamoxifen/DCA or siRNA against EGFR, even though it was decreased by DCA treatment (Supplementary Figure S1). Thus, we suggest that EGFR downregulation may not be associated with the DCA-induced metabolic shift in breast cancer cells. Because the activation of the EGFR signaling pathway contributes to tamoxifen resistance [13], we examined whether the downregulation of EGFR by inhibiting PDK sensitized the cells to tamoxifen. As shown in Figure 1D, co-treatment with tamoxifen and DCA led to a marked reduction in cell viability. The combined treatment for 72 h reduced the cell viability to less than 30% of that of the control (Figure 1E). Then, cell death in the co-treated cells was assessed using Annexin V/PI staining. Forty-eight hours after treatment, the combination of tamoxifen and DCA induced 30% cell death, compared with 15% or 8% by tamoxifen or DCA alone, respectively (Figure 1F). Previously, we reported that apoptotic cell death in breast cancer cells was caused by the loss of mitochondrial membrane potential (MMP) [14]. Thus, we tested whether loss of MMP involved in cell death induced by the co-treatment, however, there was no significant change in MMP between untreated and tamoxifen/DCA-treated cells (Supplementary Figure S2).

**Figure 2:**
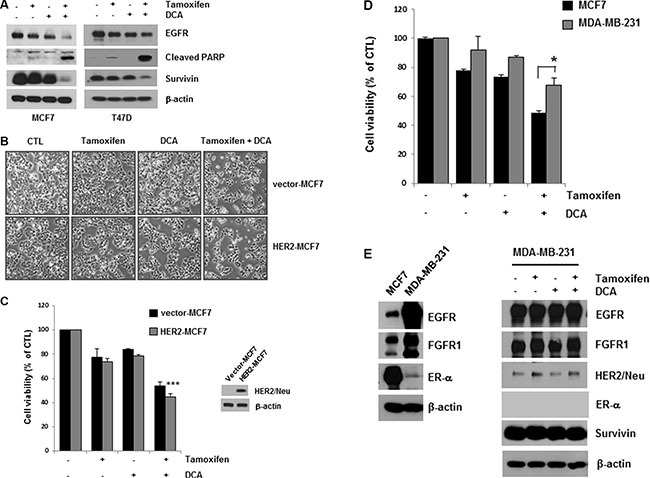
Enhancement of tamoxifen-induced cell death of ER-positive breast cancer cells by DCA treatment (**A**) MCF7 and T47D cells were treated with or without 10 μM tamoxifen and/or 20 mM DCA for 48 h, and the cell lysates were subjected to Western blotting. The blot is representative of three independent experiments. (**B** and **C**) HER2- and vector-MCF7 cells were treated with or without 10 μM tamoxifen and/or 20 mM DCA for 48 h. The cell morphological changes (B) were observed under an inverted microscope, and the images are representative of three independent experiments. Cell viability (C) was assessed using an MTT assay. Data are presented as the mean of triplicate samples, and error bars reflect the SD. ***p < 0.001 vs. untreated HER2-MCF7 cells. (**D** and **E**) MCF7 and MDA-MB-231 cells were treated with or without 10 μM tamoxifen and/or 20 mM DCA for 48 h, and the cell viability (D) was then determined. The cell lysates were analyzed by Western blotting (E). Data for the MTT assays are presented as the mean of triplicate samples, and error bars reflect the SD. Data for western blotting are representative of three independent experiments. *p < 0.05 vs. tamoxifen/DCA-treated MCF7 cells.

DCA plus tamoxifen further decreased EGFR levels in both MCF7 and T47D cells compared with that of DCA alone (Figure 2A). The cell death induced by the co-treatment was confirmed by detecting PARP cleavage, a marker of apoptosis (Figure 2A). Survivin is an anti-apoptotic molecule as well as a target of the ER [15]. The co-treatment also downregulated survivin, which may mediate apoptosis in the cells (Figure 2A). Although tamoxifen treatment decreased EGFR levels slightly in MCF7 and T47D cells, no significant increase in cell death was observed in the cells, suggesting that a critical level of EGFR is needed for the survival of breast cancer cells (Figure 2A).

Evidence from cell lines has shown that overexpression of HER2 pathways may contribute to acquired resistance to endocrine therapies [13]. To determine whether HER2 overexpression influences the cytotoxicity of tamoxifen and DCA, we examined cell viability in HER2-overexpressing MCF7 (HER2-MCF7) cells after treatment with tamoxifen and DCA. The results showed that tamoxifen and DCA significantly reduced cell viability even in HER2-MCF7 cells (Figure 2B and 2C), suggesting that DCA could enhance the tamoxifen-induced cell death in HER2-overepxressing breast cancer cells. We further evaluated the growth inhibitory effects of the co-treatment on the triple-negative breast cancer cell line MDA-MB-231. As shown in Figure 2D, MDA-MB-231 cells were less sensitive to tamoxifen and DCA than MCF7 cells. Because downregulation of EGFR was observed in ER-positive cells, we examined the effects of tamoxifen and DCA on EGFR levels in MDA-MB-231 cells. EGFR was highly expressed in MDA-MB-231 cells compared with MCF7 cells, and the levels were not significantly decreased by tamoxifen and DCA (Figure 2E). Next, we examined the cytotoxicity of tamoxifen and DCA in non-tumorigenic immortalized breast epithelial cell line MCF10A. Interestingly, the expression of EGFR in MCF10A cells was comparable to that of MDA-MB-231 cells and neither EGFR downregulation nor cell death was observed in MCF10A cells after treatment with tamoxifen and DCA (Supplementary Figure S3). These results indicate that the anti-proliferative effects of tamoxifen and DCA in breast cancer cells are dependent on EGFR downregulation.

### The combined treatment of tamoxifen and DCA induces p38 MAPK-mediated EGFR degradation

**Figure 3:**
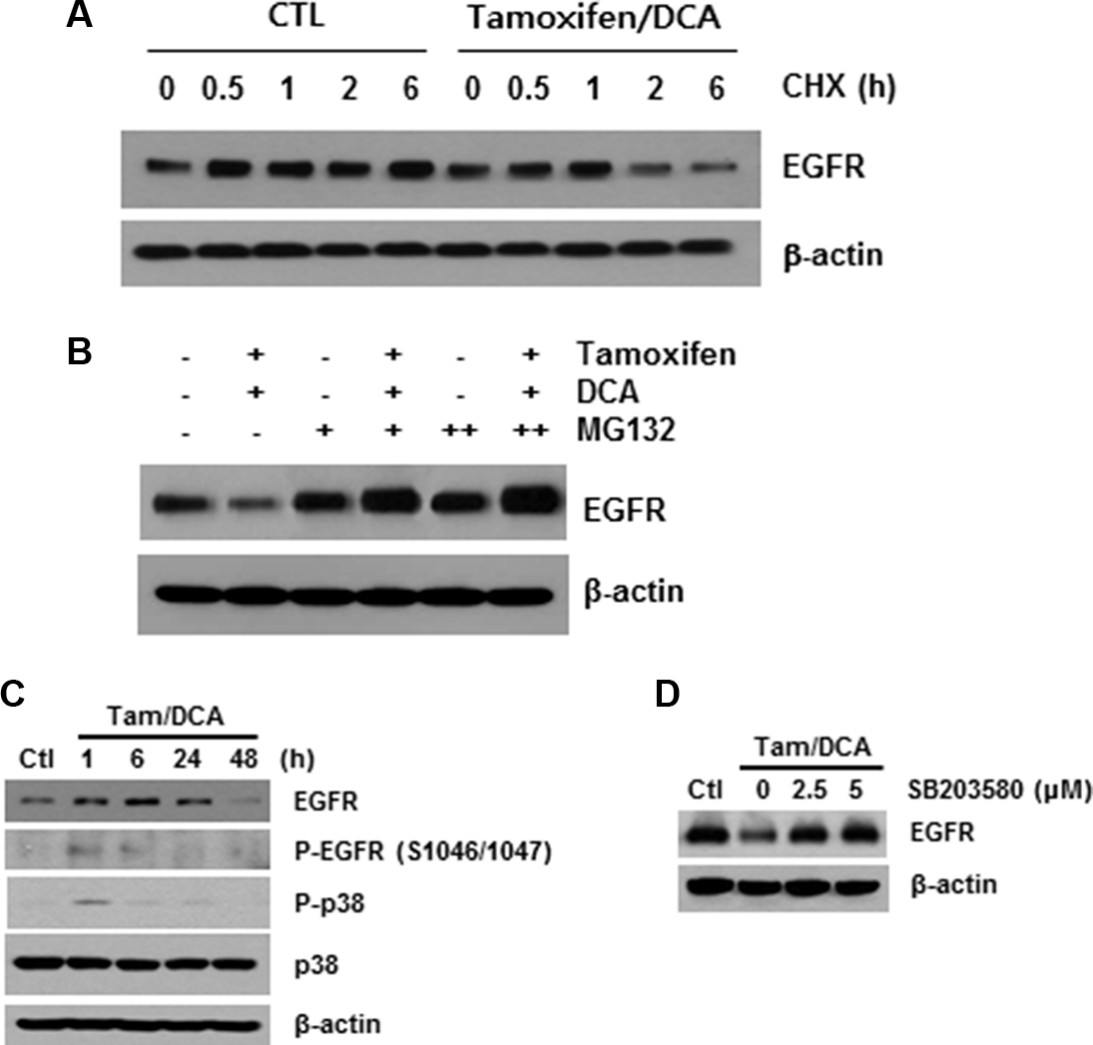
p38 MAPK-mediated EGFR degradation following combined treatment of DCA with tamoxifen (**A**) MCF7 cells were treated with 10 μM tamoxifen and 20 mM DCA for 24 h and then treated with or without 50 μg/ml cycloheximide (CHX) for the indicated times. (**B**) MCF7 cells were treated with 10 μM tamoxifen and 20 mM DCA for 24 h and then treated with or without 5 mM MG132 for 24 h. (**C**) MCF7 cells were treated with 10 μM tamoxifen and 20 mM DCA for the indicated times. (**D**) MCF7 cells were pretreated with the indicated doses of SB203580 for 1 h and then treated with 10 μM tamoxifen and 20 mM DCA for 48 h. Protein extracts were harvested and examined by Western blotting. Representative results from independent experiments conducted in triplicate with similar results are shown.

As described above, ligand binding causes rapid autophosphorylation, resulting in the removal of the EGFR from the cell surface via endocytosis into an early endosomal compartment [16]. Therefore, we next investigated the role of receptor modification in tamoxifen/DCA-mediated EGFR downregulation. After blocking protein synthesis with cycloheximide, we found that the stability of EGFR was significantly compromised in tamoxifen/DCA-treated cells compared with that of the control (Figure 3A). We then evaluated the effects of MG132, a proteasome inhibitor, on tamoxifen/DCA-induced EGFR degradation. Treatment with MG132 restored EGFR expression in tamoxifen/DCA-treated cells in a dose-dependent manner (Figure 3B).

The phosphorylation of EGFR on serine and threonine residues represents a mechanism for attenuation of the EGFR activity, and among them, the serine 1046/1047 (Ser 1046/7) phosphorylation sites are required for EGFR desensitization [17]. It has recently been reported that p38 mitogen-activated protein kinase (MAPK) induces the phosphorylation of EGFR at Ser 1046/7, which results in its degradation in cancer cells [18]. Therefore, we next examined the effects of tamoxifen and DCA on the phosphorylation of p38 MAPK in MCF7 cells. p38 MAPK was significantly phosphorylated within 1 h, and the phosphorylation was maintained for 24 h following treatment with tamoxifen and DCA (Figure 3C). Moreover, the degradation of EGFR induced by the co- treatment was significantly suppressed when the cells were pretreated with a specific p38 MAPK inhibitor, SB203580 (Figure 3D), indicating that p38 MAPK activation plays a role in tamoxifen/DCA-induced EGFR downregulation in MCF7 cells.

### EGFR inhibitors enhance the tamoxifen-induced cell death

**Figure 4:**
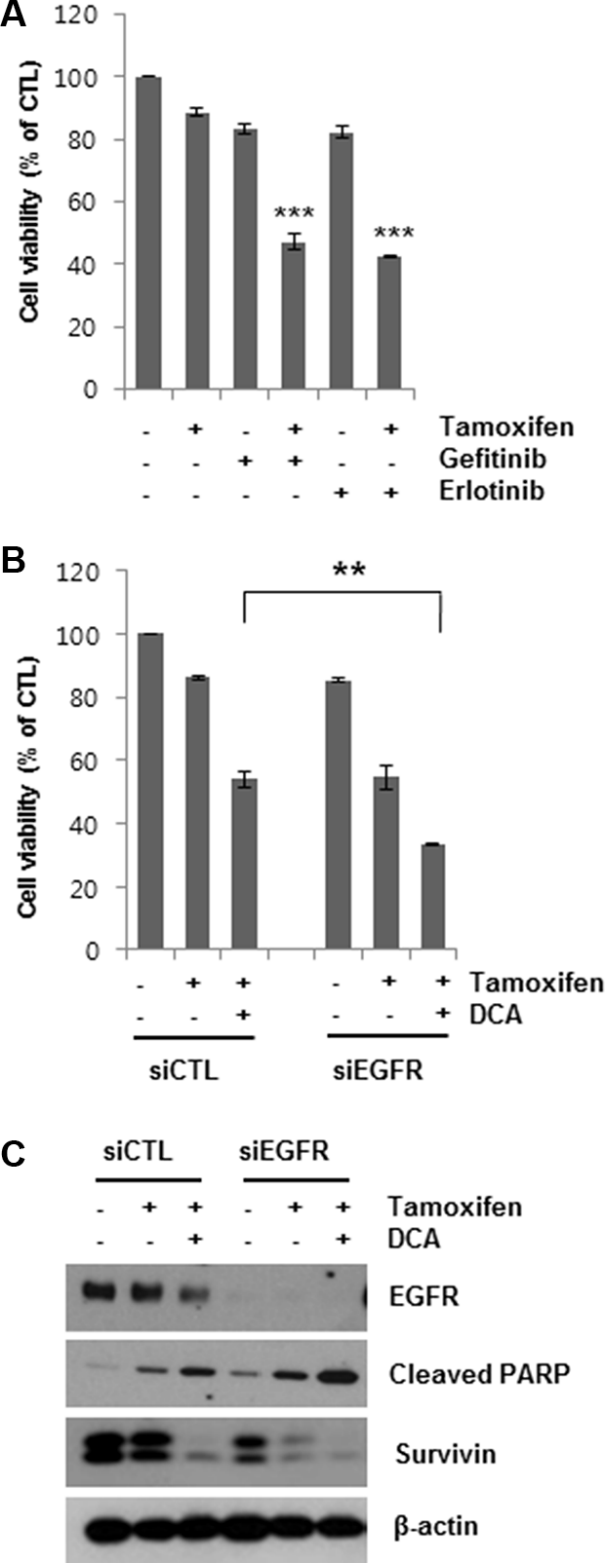
Sensitization of tamoxifen-induced cell death in MCF7 cells by EGFR inhibition (**A**) MCF7 cells were treated with 10 μM tamoxifen and 5 μM gefitinib or erlotinib for 48 h, and the cell viability was then determined. ***p < 0.001 compared with untreated group. (**B** and **C**) MCF7 cells were transfected with 60 nM siRNA targeting EGFR or negative control siRNA and then treated with or without 10 μM tamoxifen and/or 20 mM DCA for 48 h. Then, the cell viability (B) was determined. The cell lysates were analyzed by Western blotting (C). Data for the MTT assays are presented as the mean of triplicate samples, and error bars reflect the SD. C. Data for western blotting are representative of three independent experiments. **p < 0.01.

Having shown that DCA-mediated EGFR degradation could enhance tamoxifen-induced cell death in MCF7 cells, we then determined whether EGFR inhibition enhanced cell death in tamoxifen-treated cells. Gefitinib and erlotinib are selective reversible inhibitors of EGFR tyrosine kinase binding to ATP [19, 20]. Co- treatment of MCF7 cells with 5 μM gefitinib or erlotinib for 48 h markedly increased tamoxifen-induced cell death (Figure 4A). Similarly, knockdown of EGFR using siEGFR treatment enhanced tamoxifen-induced cell death (Figure 4B). The reduction in cell viability by the combined treatment of siEGFR and tamoxifen was comparable to that by DCA and tamoxifen. Moreover, siEGFR treatment further lowered EGFR levels in tamoxifen/DCA-treated cells, resulting in increased apoptotic cell death along with downregulation of survivin compared with that of the si-control (Figure 4C). These results support the conclusion that DCA sensitizes breast cancer cells to tamoxifen via downregulation of EGFR.

### c-myc and Nanog expression are associated with the cytotoxic effects of DCA in tamoxifen-treated MCF7 cells

**Figure 5:**
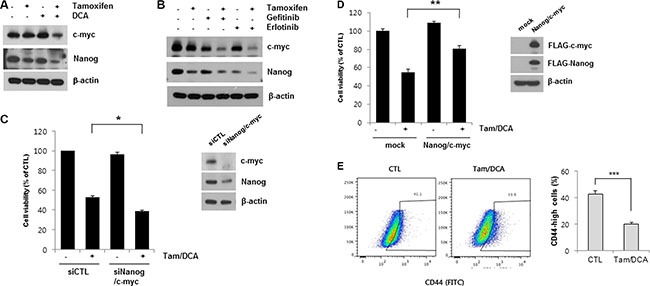
c-myc and Nanog expression is associated with cytotoxicity induced by the combined treatment of DCA and tamoxifen in MCF7 cells (**A**) MCF7 cells were treated with or without 10 μM tamoxifen or/and 20 mM DCA for 48 h. (**B**) MCF7 cells were treated with 10 μM tamoxifen and 5 μM gefitinib or erlotinib for 48 h. The cell lysates were subjected to Western blotting. Data are representative of three independent experiments. (**C**) MCF7 cells were transiently transfected with Nanog siRNA and c-myc siRNA or control siRNAs and then treated with or without 10 μM tamoxifen and 20 mM DCA for 48 h. (**D**) MCF7 cells were transiently transfected with pcDNA-FLAG-Nanog and pcDNA-FLAG-c-myc or an empty vector and then treated with or without 10 μM tamoxifen and 20 mM DCA for 48 h. (**E**) MCF7 cells were treated with or without 10 μM Tamoxifen and 20 mM DCA for 48 h and then analyzed CD44 positive cell population on flow cytometry. Data are representative of three independent experiments. Data are presented as the mean of triplicate samples, and error bars reflect the SD. *p < 0.05, **p < 0.01,***p < 0.001.

EGFR activation in cancer has been shown to increase various transcription factors that may affect the type and the duration of EGFR signaling [21]. Among them, Nanog and c-myc have a pleiotropic role in tumorigenesis, including resistance to standard therapy in breast carcinoma [22, 23]. To further investigate the anti-tumor activities mediated by EGFR downregulation in breast cancer cells, we examined the expression of c-myc and Nanog in MCF7 cells after co-treatment with tamoxifen and DCA. The levels of c-myc and Nanog were significantly decreased in the cells co-treated with tamoxifen and DCA (Figure 5A). Similarly, the expressions of both proteins were suppressed by tamoxifen in combination with gefitinib or erlotinib (Figure 5B), indicating that EGFR expression is needed to maintain the expression of c-myc and Nanog in tamoxifen-treated MCF7 cells. Nevertheless, there was little or no effect of siEGFR on the expression of c-myc and Nanog, indicating that EGFR downregulation is necessary but not sufficient to decrease the expression of the proteins (Supplementary Figure S4). To determine whether the reduced expression of these two proteins is involved in the cytotoxic effects of tamoxifen and DCA in MCF7 cells, we tested the effects of siRNAs against c-myc and Nanog in cells exposed to tamoxifen/DCA. Knockdown of c-myc and Nanog significantly sensitized cells to tamoxifen/DCA compared with the controls (Figure 5C). Conversely, overexpression of FLAG-c-myc and FLAG-Nanog by transfection of cells with FLAG-c-myc and FLAG-Nanog vectors significantly protected cells from tamoxifen/DCA-induced cytotoxicity (Figure 5D). Taken together, our data suggest that EGFR downregulation by a combination of tamoxifen and DCA may induce cell death in MCF7 cells partially through the inhibition of c-myc and Nanog expression. It has been known that self-renewal genes, such as c-myc and Nanog are associated with cancer stem-like cell properties [24]. To test whether co-treatment with tamoxifen and DCA inhibits breast cancer stem-like cells, we performed flow cytometry analysis to estimate the proportion of stem-like cell subpopulation in MCF7 cells based on the expression CD44. After the co-treatment, CD44-high population in MCF7 cells was reduced from 42.5% to 19.9% (Figure 5E). This finding raised the possibility that DCA plus tamoxifen might inhibit cancer stem-like cell capability in breast cancer cells.

### The combined treatment with DCA and tamoxifen can overcome tamoxifen resistance in breast cancer cells

**Figure 6:**
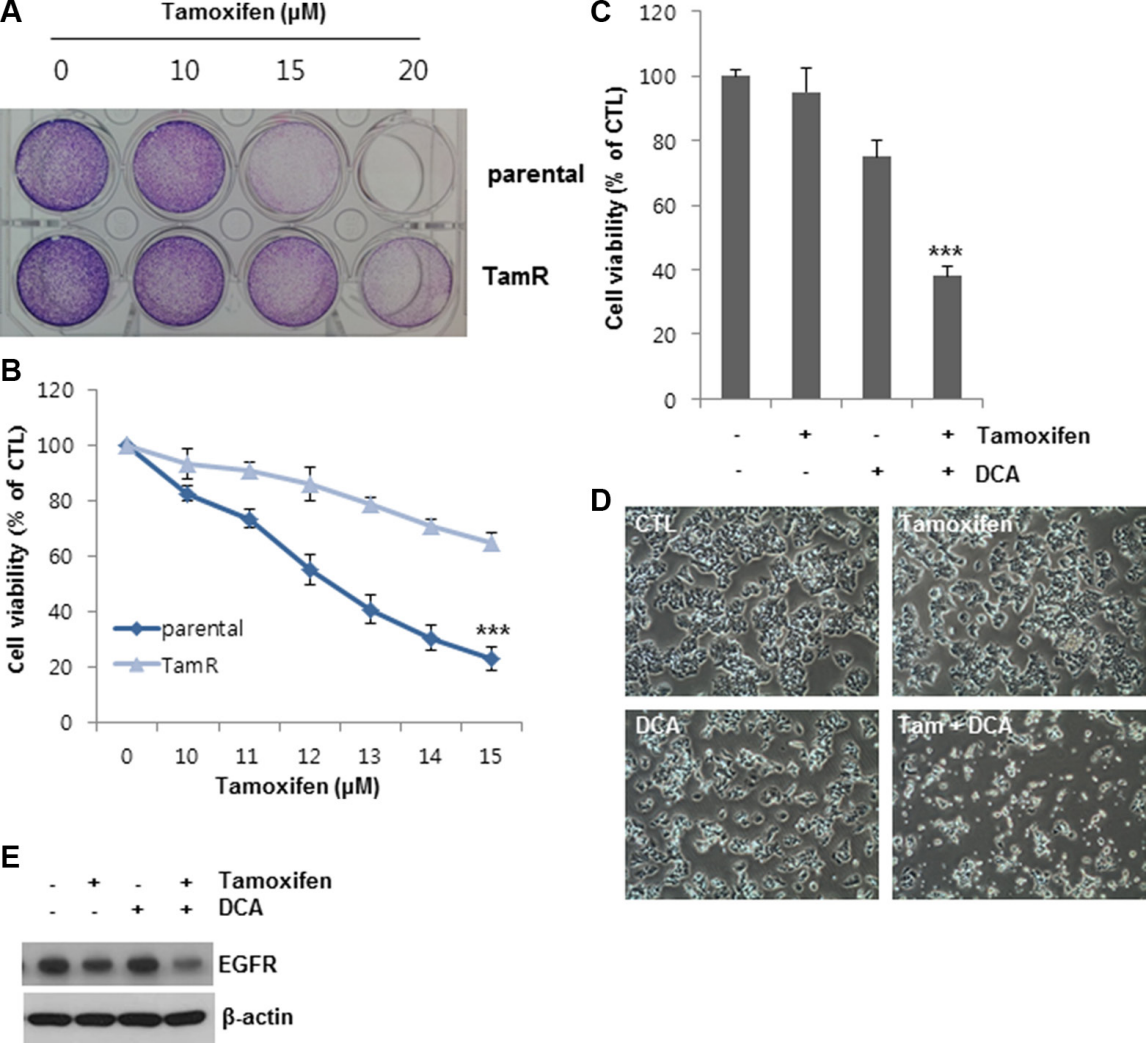
DCA sensitizes tamoxifen-resistant MCF7 cells to tamoxifen (**A** and **B**) MCF7 parental cells and tamoxifen resistant (TamR) MCF7 cells were treated with the indicated concentrations of tamoxifen for 48 h. After incubation of the cells in medium containing MTT, the media were removed, and images (A) were obtained before solubilizing the cells. The data for staining are representative of three independent experiments. Data for MTT assays (B) are presented as the mean of triplicate samples, and error bars reflect the SD. ***p < 0.001 vs. tamoxifen-treated parental group. (**C** and **D**) TamR MCF7 cells were treated with or without 10 μM tamoxifen and/or 20 mM DCA for 48 h. Cell viability (C) was assessed by an MTT assay. Data for the MTT assays are presented as the mean of triplicate samples, and error bars reflect the SD. ***p < 0.001 vs. untreated group. The cell morphological changes (D) were observed under an inverted microscope, and the images are representative of three independent experiments. Data for the cell images are representative of three independent experiments. (**E**) TamR MCF7 cells were treated with or without 10 μM tamoxifen and/or 20 mM DCA for 48 h. The cell lysates were analyzed by Western blotting. Data are representative of three independent experiments.

To further confirm the observed effects of DCA on tamoxifen-induced cell death, we established tamoxifen-resistant (TamR) MCF7 cells by tamoxifen treatment over a long period of time. Compared to the untreated cells, the cell viability of the MCF7 and TamR MCF7 cells was 20 and 60%, respectively, following treatment with 13 μM tamoxifen, indicating that the TamR MCF7 cells were less sensitive to the same concentration of tamoxifen than the MCF7 parental cells (Figure 6A and 6B). DCA alone inhibited the growth of TamR MCF7 cells by approximately 25% of that of the control (Figure 6C and 6D). In contrast, co-treatment of DCA with tamoxifen suppressed the cell growth by more than 60% compared to the control (Figure 6C and 6D). The EGFR levels in TamR cells were also downregulated by the co-treatment (Figure 6E). These results suggest that DCA could overcome tamoxifen resistance in breast cancer cells by EGFR downregulation at the protein level.

## DISCUSSION

As the understanding of the metabolic phenotype of tumor cells has grown, targeting the metabolic differences between tumor and normal cells has been proposed as a novel anticancer strategy. Despite the increase in potential anticancer drugs that target metabolic processes of cancer cells, elucidation of how cells respond to the drugs will be necessary to successfully target cancer metabolism. Here, we demonstrated that DCA inhibits not only PDK activity but also EGFR expression in breast cancer cells. The combined treatment of DCA and tamoxifen induced proteasome-dependent degradation of EGFR proteins in breast cancer cells via p38 MAPK activation. Our results indicate that EGFR downregulation plays a key role in apoptotic cell death induced by a combination of DCA and tamoxifen. To our knowledge, this is the first report showing that DCA sensitizes breast cancer cells to tamoxifen-induced cell death via EGFR downregulation.

In ER-positive breast cancer cells that have developed endocrine resistance, ER expression may be directly suppressed by enhanced growth factor receptor signaling due to overexpression of EGFR and HER2, which subsequently activate MAPK and inhibit ER transcription [25]. In addition, resistance to tamoxifen in breast cancer cells has been related to overexpression of EGFR and high levels of phosphorylated extracellular activated kinase 1/2 [2]. Thus, the strategy of combining an EGFR inhibitor with an endocrine agent is of sufficient interest to warrant further study for breast cancer therapy. In this study, we found that downregulation of EGFR in DCA-treated breast cancer cells enhanced the sensitivity of the cells to tamoxifen. EGFR downregulation by co- treatment of DCA with tamoxifen has been confirmed in several ER-positive breast cancer cell lines, including MCF7, T47D and BT474 (Figure 2A and not shown). In addition to the HER2-amplified breast cancer cell line BT474, HER2-MCF7 cells also showed EGFR downregulation by DCA/tamoxifen, suggesting that the combination treatment will be applicable to HER2-postive breast cancer. Unfortunately, a triple-negative breast cancer cell line, MDA-MB-231, which is known to overexpress EGFR [26], showed a refractory phenotype as well as no obvious change in EGFR levels in response to the co-treatment. One possibility is that the amplification of EGFR in MDA-MB-231 cells overwhelms its downregulation by co-treatment of DCA with tamoxifen. The other explanation is that the signaling pathway(s) that mediate the EGFR downregulation are blocked by an unknown mechanism in MDA-MB-231 cells. Further investigation will be needed to elucidate the underlying mechanism for maintenance of EGFR expression in response to co-treatment of DCA and tamoxifen in ER-negative breast cancer cells.

Four isoforms of PDK are known, each active in response to different intracellular and extracellular conditions. PDK1 is activated by hypoxia [27]; PDK2 is activated by the PDH products acetyl CoA and NADH [28]; PDK3 is activated by ATP [29]; and PDK4 is regulated transcriptionally by hormonal signals [30]. PDK2 possesses the greatest activity of phosphorylating pyruvate dehydrogenase complex followed by PDK4, PDK1 and PDK3 [12]. PDK2 is most susceptible to inhibition by DCA because of its ubiquitous expression [31]. Although we detected the expression of four isoforms in MCF7 cells (Figure 1C), the functional differences between PDK isoforms in breast cancer still remains elusive. Further investigating whether signaling pathways suppressing particular isoform of PDK lead to EGFR downregulation will be interest. PDK is known to function in the mitochondria, and several studies demonstrated that EGFR also translocates to the mitochondria after EGF stimulation [32, 33]. Furthermore, it has been suggested that the interaction of EGFR with PDK in the mitochondrial matrix plays an important role in EGFR-induced tumor growth in glioblastoma multiforme [34]. Here, we found that the blockade of PDK activity by an inhibitor or silencing decreased total cellular EGFR levels in tamoxifen-treated breast cancer cells. Further studies are needed to determine whether only mitochondrial-associated EGFR is subject to degradation by co-treatment with DCA and tamoxifen in breast cancer cells. In general, exposure of cells to EGF caused rapid autophosphorylation, including tyrosine (Tyr) 1045, which provides a docking site for the ubiquitin ligase c-Cbl, thus resulting in the ubiquitination of the EGFR and removal of the EGFR via endocytosis from the cell surface into an early endosomal compartment [35]. However, co-treatment with DCA and tamoxifen had no effect on the phosphorylation of EGFR at Tyr 1045 (data not shown). Instead, we observed the phosphorylation of EGFR at Ser 1046/7 by DCA and tamoxifen, which was blocked by a specific p38 MAPK inhibitor, SB203580. It has been suggested that p38 MAPK plays a pivotal role in both the internalization and the degradation of EGFR [18]; however, further investigation is required to elucidate how EGFR phosphorylation at serine residues causes its downregulation and to identify the upstream mediator of p38 MAPK activation after DCA/tamoxifen treatment.

Nanog and c-myc have been identified as transcription factors, and their role in maintaining self-renewal in embryonic stem cells has been established in previous studies [22, 36]. A recently proposed mechanism of intrinsic resistance to endocrine therapy posits the existence of a specialized subset of cancer cells called tumor-initiating cells (TICs) [37]. TICs have the capacity to self-renew and to generate new tumors that consist entirely of clonally derived cell types present in the parental tumor. Here, we demonstrated that DCA decreased the expression of c-myc and Nanog in tamoxifen-treated MCF7 cells. Moreover, the expression of these proteins was related to the cytotoxic effects of the combined treatment of DCA and tamoxifen. Therefore, DCA can be exploited to expand the therapeutic potential of tamoxifen by suppressing the development of intrinsic resistance in breast cancer therapy.

In conclusion, our results revealed that DCA sensitized ER-positive breast cancer cells to tamoxifen by decreasing EGFR levels. Treatment with DCA and tamoxifen inhibited the expression of self-renewal genes and the survival of tamoxifen-resistant MCF7 cells. Therefore, we propose that DCA may be an effective therapeutic agent for treating tamoxifen-resistant breast cancer. Moreover, combination strategies with DCA may be useful for enhancing the treatment efficacy of other cytotoxic chemotherapies or targeted therapies. Further experiments, including animal studies and clinical trials, should be carried out in the future.

## MATERIALS AND METHODS

### Cell culture and reagents

MCF7, T47D, and MDA-MB-231 human breast cancer cells were purchased from the American Type Culture Collection (Rockville, MD, USA) and were grown in the recommended growth medium (Invitrogen, Carlsbad, CA, USA). HER2-overexpressing MCF7 (HER2-MCF7) and control vector cells (vector-MCF7) were kindly provided by Dr. Incheol Shin (Hanyang University, Seoul, Korea). Tamoxifen-resistant (TamR) MCF7 cells were developed by culturing MCF7 cells in the presence of 10 μM tamoxifen for more than 6 months. As a control, parental cells were cultured for the same amount of time in regular media. Following the establishment of resistance, cells were passaged for no more than 3 months. For experiments involving tamoxifen treatments, cells were routinely cultured in phenol red-free Dulbecco's modified Eagle's medium plus 10% charcoal-stripped fetal bovine serum just before the treatments. Antibodies against EGFR, phospho-EGFR (S1046/1047), FGFR1, FGFR4, PDK1, PDH, survivin, cleaved PARP, p38, phospho-p38, and Nanog were acquired from Cell Signaling Technology (Danvers, MA, USA). Antibodies against HER2/Neu, ER-α and c-myc, siRNAs targeting EGFR, Nanog, PDK4, and c-myc and the negative control (scrambled) siRNAs were acquired from Santa Cruz Biotechnology (Dallas, TX, USA). Antibodies against PDK2 and PDK3 were acquired from Thermo Fisher Scientific (Waltham, MA, USA). The β-actin antibody, FLAG antibody, tamoxifen, DCA, cycloheximide and MG132 were purchased from Sigma-Aldrich (St. Louis, MO, USA). The phospho-PDH (S293) antibody and SB203580 were from BD Biosciences Pharmingen (San Diego, CA, USA), and gefitinib and erlotinib were obtained from Selleck Chemicals (London, ON, Canada).

### Transfections and treatments

Cells were transfected with the target siRNA (50 nM) using Lipofectamine RNAiMAX (Invitrogen) as described by the manufacturer. Cells were transfected with 1 μg FLAG-c-myc pcDNA 3.1 and FLAG-Nanog-pcDNA 3.1 using Lipofectamine 2000 as described by the manufacturer. After 6 h, cells were treated with tamoxifen and/or DCA for 24–48 h and then analyzed as described elsewhere.

### Measurement of cell viability

Cell viability was determined by measuring the mitochondrial conversion of 3-(4,5-dimethylthiazolyl-2)-2,5-diphenyltetrazolium bromide (MTT) to a colored product. Cells were treated as indicated, and the medium was exchanged with serum-free medium containing 1 mM MTT. After 2 h of incubation at 37°C, cells were solubilized in DMSO. The amount of formazan, the converted form of MTT, was determined by measuring absorbance at 595 nm.

### Evaluation of apoptosis

Apoptosis was determined by fluorescence-activated cell sorting analysis using an Annexin V-FITC apoptosis kit (BioVision, Milpitas, CA, USA) as directed by the manufacturer. Briefly, after treatment, cells were treated with trypsin and then resuspended in binding buffer (10 mM HEPES/NaOH, pH 7.4, 140 mM NaCl, 2.5 mM CaCl_2_) including Annexin V-FITC and propidium iodide. After incubation for 15 min, cell fluorescence was analyzed by flow cytometry. Cell death was measured as the percentage of cells in the Annexin V and PI positive population.

### Western blotting

Cells were harvested and lysed in RIPA buffer (50 mM Tris-HCl pH 7.5, 150 mM NaCl, 1% Nonidet P40, 0.5% sodium deoxycholate, and 0.1% SDS) supplemented with a protease/phosphatase inhibitor cocktail (Roche, Mannheim, Germany). Equal amounts of proteins (20– 50 μg) were separated by SDS-PAGE and transferred to a nitrocellulose membrane. Membranes were blocked by incubating with 5% skim milk in Tris-buffered saline for 1 h and were then incubated overnight with the appropriate primary antibodies. Membranes were incubated with HRP-conjugated secondary antibody for 1 h. Immunoreactive proteins were visualized using enhanced chemiluminescence reagents (Amersham Biosciences, Little Chalfont, UK).

### Detection of CD44 positive cell population

Cells were stained with antibodies at 1: 100 dilution in PBS for 15 minutes. The antibodies used were as follows; FITC-CD44 and FITC-conjugated mouse IgG isotype control antibodies were obtained from BD Biosciences Pharmingen. Labeled cells were analyzed on flow cytometry. Populations of CD44 positive cells were determined by the intensity of FITC.

### Statistical analysis

All data presented are representative of at least two separate experiments. Comparisons between groups were analyzed using Student's *t*-test. Asterisks (***p < 0.001, **p < 0.01, *p < 0.05) indicate statistical significance.

## SUPPLEMENTARY MATERIALS FIGURES



## References

[1] Vander Heiden MG, Cantley LC, Thompson CB (2009). Understanding the Warburg effect: the metabolic requirements of cell proliferation. Science.

[2] Zhao Y, Butler EB, Tan M (2013). Targeting cellular metabolism to improve cancer therapeutics. Cell Death Dis.

[3] Vander Heiden MG (2011). Targeting cancer metabolism: a therapeutic window opens. Nat Rev Drug Discov.

[4] Bonnet S, Archer SL, Allalunis-Turner J, Haromy A, Beaulieu C, Thompson R, Lee CT, Lopaschuk GD, Puttagunta L, Bonnet S, Harry G, Hashimoto K, Porter CJ (2007). A mitochondria-K+ channel axis is suppressed in cancer and its normalization promotes apoptosis and inhibits cancer growth. Cancer Cell.

[5] Kankotia S, Stacpoole PW (2014). Dichloroacetate and cancer: new home for an orphan drug?. Biochim Biophys Acta.

[6] Osborne CK (1998). Tamoxifen in the treatment of breast cancer. N Engl J Med.

[7] Johnston SR (2010). New strategies in estrogen receptor-positive breast cancer. Clin Cancer Res.

[8] Peto R, Boreham J, Clarke M, Davies C, Beral V (2000). UK and USA breast cancer deaths down 25% in year 2000 at ages 20–69 years. Lancet.

[9] Early Breast Cancer Trialists' Collaborative Group (EBCTCG) (2005). Effects of chemotherapy and hormonal therapy for early breast cancer on recurrence and 15-year survival: an overview of the randomised trials. Lancet.

[10] Davies C, Godwin J, Gray R, Clarke M, Cutter D, Darby S, McGale P, Pan HC, Taylor C, Wang YC, Dowsett M, Ingle J, Early Breast Cancer Trialists' Collaborative Group (EBCTCG) (2011). Relevance of breast cancer hormone receptors and other factors to the efficacy of adjuvant tamoxifen: patient-level meta-analysis of randomised trials. Lancet.

[11] Velpula KK, Bhasin A, Asuthkar S, Tsung AJ (2013). Combined targeting of PDK1 and EGFR triggers regression of glioblastoma by reversing the Warburg effect. Cancer Res.

[12] Saunier E, Benelli C, Bortoli S (2016). The pyruvate dehydrogenase complex in cancer: An old metabolic gatekeeper regulated by new pathways and pharmacological agents. Int J Cancer.

[13] Osborne CK, Schiff R (2011). Mechanisms of endocrine resistance in breast cancer. Annu Rev Med.

[14] Yun SM, Woo SH, Oh ST, Hong SE, Choe TB, Ye SK, Kim EK, Seong MK, Kim HA, Noh WC, Lee JK, Jin HO, Lee YH (2016). Melatonin enhances arsenic trioxide-induced cell death via sustained upregulation of Redd1 expression in breast cancer cells. Mol Cell Endocrinol.

[15] Zhu J, Lu X, Hua KQ, Sun H, Yu YH, Feng YJ (2012). Oestrogen receptor α mediates 17β-estradiol enhancement of ovarian cancer cell motility through up-regulation of survivin expression. Arch Gynecol Obstet.

[16] Arteaga CL (2002). Epidermal growth factor receptor dependence in human tumors: more than just expression?. Oncologist.

[17] Sorkin A, Goh LK (2009). Endocytosis and intracellular trafficking of ErbBs. Exp Cell Res.

[18] Adachi S, Shimizu M, Shirakami Y, Yamauchi J, Natsume H, Matsushima-Nishiwaki R, To S, Weinstein IB, Moriwaki H, Kozawa O (2009). (-)-Epigallocatechin gallate downregulates EGF receptor via phosphorylation at Ser1046/1047 by p38 MAPK in colon cancer cells. Carcinogenesis.

[19] Ciardiello F, Caputo R, Bianco R, Damiano V, Pomatico G, De Placido S, Bianco AR, Tortora G (2000). Antitumor effect and potentiation of cytotoxic drugs activity in human cancer cells by ZD-1839 (Iressa), an epidermal growth factor receptor-selective tyrosine kinase inhibitor. Clin Cancer Res.

[20] Pollack VA, Savage DM, Baker DA, Tsaparikos KE, Sloan DE, Moyer JD, Barbacci EG, Pustilnik LR, Smolarek TA, Davis JA, Vaidya MP, Arnold LD, Doty JL (1999). Inhibition of epidermal growth factor receptor-associated tyrosine phosphorylation in human carcinomas with CP-358,774: dynamics of receptor inhibition *in situ* and antitumor effects in athymic mice. J Pharmacol Exp Ther.

[21] Normanno N, De Luca A, Bianco C, Strizzi L, Mancino M, Maiello MR, Carotenuto A, De Feo G, Caponigro F, Salomon DS (2006). Epidermal growth factor receptor (EGFR) signaling in cancer. Gene.

[22] Iv Santaliz-Ruiz LE, Xie X, Old M, Teknos TN, Pan Q (2014). Emerging role of nanog in tumorigenesis and cancer stem cells. Int J Cancer.

[23] Chen Y, Olopade OI (2008). MYC in breast tumor progression. Expert Rev Anticancer Ther.

[24] Hou ZJ, Luo X, Zhang W, Peng F, Cui B, Wu SJ, Zheng FM, Xu J, Xu LZ, Long ZJ, Wang XT, Li GH, Wan XY (2015). Flubendazole, FDA-approved anthelmintic, targets breast cancer stem-like cells. Oncotarget.

[25] Creighton CJ, Hilger AM, Murthy S, Rae JM, Chinnaiyan AM, El-Ashry D (2006). Activation of mitogen-activated protein kinase in estrogen receptor alpha-positive breast cancer cells *in vitro* induces an *in vivo* molecular phenotype of estrogen receptor alpha-negative human breast tumors. Cancer Res.

[26] Biswas DK, Cruz AP, Gansberger E, Pardee AB (2000). Epidermal growth factor-induced nuclear factor kappa B activation: A major pathway of cell-cycle progression in estrogen-receptor negative breast cancer cells. Proc Natl Acad Sci U S A.

[27] Kim JW, Tchernyshyov I, Semenza GL, Dang CV (2006). HIF-1-mediated expression of pyruvate dehydrogenase kinase: a metabolic switch required for cellular adaptation to hypoxia. Cell Metab.

[28] Hiromasa Y, Hu L, Roche TE (2006). Ligand-induced effects on pyruvate dehydrogenase kinase isoform 2. J Biol Chem.

[29] Kato M, Chuang JL, Tso SC, Wynn RM, Chuang DT (2005). Crystal structure of pyruvate dehydrogenase kinase 3 bound to lipoyl domain 2 of human pyruvate dehydrogenase complex. EMBO J.

[30] Kwon HS, Huang B, Unterman TG, Harris RA (2004). Protein kinase B-alpha inhibits human pyruvate dehydrogenase kinase-4 gene induction by dexamethasone through inactivation of FOXO transcription factors. Diabetes.

[31] Bowker-Kinley MM, Davis WI, Wu P, Harris RA, Popov KM (1998). Evidence for existence of tissue-specific regulation of the mammalian pyruvate dehydrogenase complex. Biochem J.

[32] Dasari VR, Velpula KK, Alapati K, Gujrati M, Tsung AJ (2012). Cord blood stem cells inhibit epidermal growth factor receptor translocation to mitochondria in glioblastoma. PLoS One.

[33] Demory ML, Boerner JL, Davidson R, Faust W, Miyake T, Lee I, Hüttemann M, Douglas R, Haddad G, Parsons SJ (2009). Epidermal growth factor receptor translocation to the mitochondria: regulation and effect. J Biol Chem.

[34] Velpula KK, Bhasin A, Asuthkar S, Tsung AJ (2013). Combined targeting of PDK1 and EGFR triggers regression of glioblastoma by reversing the Warburg effect. Cancer Res.

[35] Massie C, Mills IG (2006). The developing role of receptors and adaptors. Nat Rev Cancer.

[36] Chappell J, Dalton S (2013). Roles for MYC in the establishment and maintenance of pluripotency. Cold Spring Harb Perspect Med.

[37] Wei W, Lewis MT (2015). Identifying and targeting tumor-initiating cells in the treatment of breast cancer. Endocr Relat Cancer.

