# Supplementary material for: Dichloroacetate potentiates tamoxifen-induced cell death in breast cancer cells via downregulation of the epidermal growth factor receptor
Source: Oncotarget. 2016 Aug 1;7(37):59809–19. doi: 10.18632/oncotarget.10999 (PMC5312350; doi:10.18632/oncotarget.10999)
Supplement: Supplementary file 1 [file oncotarget-07-59809-s001.pdf]

Dichloroacetate potentiates tamoxifen-induced cell death in breast cancer cells via downregulation of the epidermal growth factor receptor

Supplementary Materials

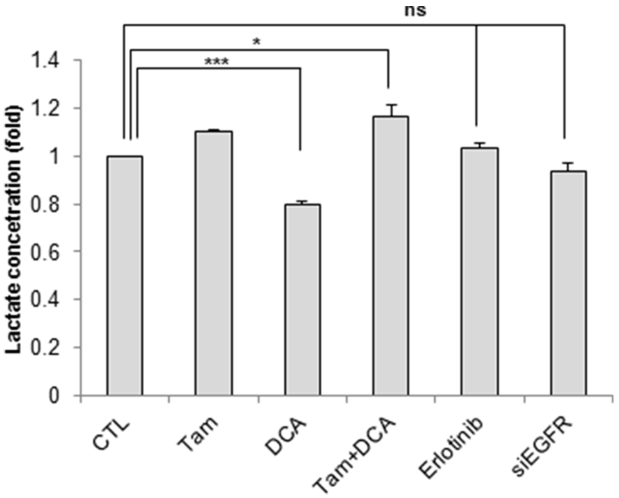

Supplementary Figure S1: EGFR downregulation is not related to lactate secretion in MCF7 Cells. Lactate Concentrations in the supernatant of MCF7 Cells were measured using L-lactate assay kit (Bio Assay Systems, Haymard, CA, USA) after treatment with 10 μm tamoxifen, 20 mM DCA, 5 μM erlotinib or siEGFR for 48 h. *$p < 0.05$; ***$p < 0.001$; ns, $p > 0.05$.

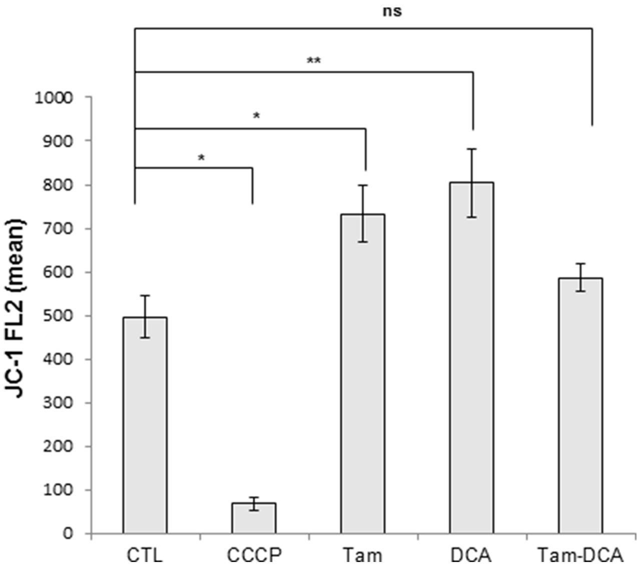

Supplementary Figure S2: The loss of mitochondrial membrane potential (MMP) is nor associated with cell death induced by co-treatment with tamoxifen and DCA in MCF7 cells. MMP was assessed using a mitochondria staing kit (MitoPT, Immunohistochemistry Technologies, Blllmington, MN, USA) based on 5, 50, 6, 60-tetrachloro-1, 10, 3, 30-tetraethylbenzimidazol-carbocyanine iodide (JC-1), which is voltage-sensitive lipophilic cationic fluorescence probe. Cells were exposed to 10 μM tamoxifen, 20 mM DCA, or both 10 μM tamoxifen and 20 mM DCA for 48 h and then MMP was analyzed by flow cytometry using JC-1. Ten μM carbonyl cyanide 3-chlorophenylhydrazone (CCCP) was added as a positive control. *$P < 0.05$; **$P < 0.01$ ns, $P > 0.05$.

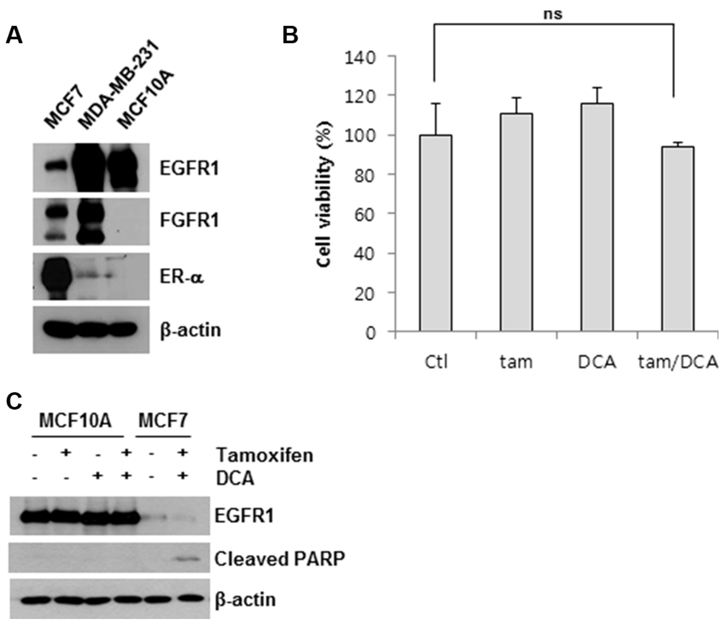

Supplementary Figure S3: MCF10A cells are less sensitive to tamoxifen and DCF than MCF7 cells. (**A**) The cell lysates were analyzed by Western blotting. (**B**) MCF10A cells were treated with or without 10 μM tamoxifen and/or 20 mM DCA for 48 h, and the cell viability was then determined using an MTT assay. (**C**) MCF10A and MCF7 cells were treated with or without 10 μM tamoxifen and/or 20 mM DCA for 48 h, and the cell lysates were analyzed by Western blotting. ns, $p > 0.05$

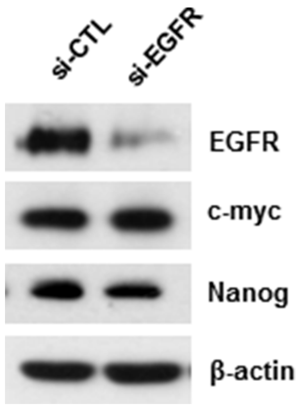

Supplementary Figure S4: Effect of siEGFR on the expression of c-myc and Nanog in MCF7 cells. MCF7 cell were treated with siRNA against EGFR for 48 h, and cell lysates were subjected to Western blotting. The blot is representative of two independent experiments.
